# Mercury-associated glomerulonephritis: a retrospective study of 35 cases in a single Chinese center

**DOI:** 10.1186/s12882-019-1413-z

**Published:** 2019-06-20

**Authors:** Ai-bo Qin, Tao Su, Su-xia Wang, Fan Zhang, Fu-de Zhou, Ming-hui Zhao

**Affiliations:** 1 Renal Division, Department of Medicine, Peking University First Hospital, Institute of Nephrology, Peking University, Key Laboratory of Renal Disease, Ministry of Health of China, Key Laboratory of CKD Prevention and Treatment, Ministry of Education of China, Beijing, 100034 China; 2 Electron Microscopy Laboratory, Peking University First Hospital, Beijing, China; 3 Peking-Tsinghua Center for Life Sciences, Beijing, China

**Keywords:** Mercury, Glomerulonephritis, Membranous nephropathy, Minimal change disease

## Abstract

**Background:**

Long-term exposure of mercury may induce glomerulonephritis. Clinical and pathological features of mercury-associated glomerulonephritis are not fully clear. This study retrospectively analyzed 35 cases of mercury-associated glomerulonephritis in a single Chinese center.

**Methods:**

Thirty-five patients of mercury-associated glomerulonephritis were enrolled. Clinical data on diagnosis and during follow-up were collected. Plasma anti-phospholipase A2 receptor (PLA2R) antibody, glomerular PLA2R and glomerular IgG subclasses deposition were detected in the cases with membranous nephropathy (MN).

**Results:**

Mercury exposure was caused by skin lighting cream (20 patients), mercury-containing pills (9 patients), hair-dyeing agents (4 patients), and unidentified reasons (2 patients). All patients presented with proteinuria and normal renal function. The median of urinary protein was 4.6 (range 1.6~19.7) g/24 h. Twenty-two patients (62.9%) had nephrotic syndrome. Renal histopathology showed minimal change disease (MCD) in 21 patients (60.0%), MN in 13 (37.1%) and focal segmental glomerular sclerosis (FSGS) in 1 patient (2.9%). The proportion of MCD increased along with urinary mercury concentration (*P* = 0.024). In 13 cases of MN, all patients were negative for plasma anti-PLA2R antibody and glomerular PLA2R antigen. IgG1 (61.5%) and IgG4 (46.2%) deposits were noted along the glomerular capillary loops. Among the 16 patients received mercury detoxification monotherapy, 14 patients received 4.5 ± 2.8 (range 1~12) rounds of regimen and achieved complete remission in 4.5 (range 0.3~23.0) months, 2 patients stayed no remission.

**Conclusions:**

MCD was the most common pathological type of mercury-associated glomerulonephritis, followed by MN. The proportion of MCD increased along with the increase of urinary mercury concentration. Most patients could achieve complete remission after mercury detoxification.

## Background

Mercury is a kind of heavy metal with nephrotoxicity, which exists in metallic, inorganic, and organic forms [1]. As early as 1818, there has been a report of proteinuria caused by mercury compounds [2]. Mercury can be absorbed into the body through inhalation, ingestion, skin and injection. Mercury poisoning is mainly due to fish consumption, dental amalgams and vaccines [3]. The application of mercury-containing skin lighting cream [4], as well as the improper use of Chinese traditional medicine, could also lead to the accumulation of mercury in the body [5]. Various forms of mercury and its compounds can cause damage to a number of organs, in particular, kidneys, nervous system and gastrointestinal tract [3].

In recent years, mercury-associated kidney disease has apparently decreased in developed countries, but mercury exposure is still not rare in developing countries [6]. Since lack of specific clinical manifestations, mercury-associated glomerulonephritis is often misdiagnosed as primary glomerular diseases. Moreover, the pathological spectrum and mechanism of mercury-associated glomerulonephritis has not been fully elucidated. The current study retrospectively reviewed 35 cases of mercury-associated glomerulonephritis over a 6-year period to analyze the clinical and pathological features, treatment and prognosis of this disease.

## Methods

### Patient recruitment

Thirty-five patients of mercury-associated glomerulonephritis between January 2010 and December 2015 in Peking University First Hospital were recruited retrospectively. These patients were met the following inclusion criteria: *(1)* the patient had a definite history of exposure to mercury-containing preparations, whose serum mercury was > 2.5 μg/L and spot urinary mercury > 10 μg/L; *(2)* the kidney abnormalities confirmed by clinical and/or pathological parameters, such as proteinuria, hematuria, abnormal kidney function. Patients with any history of primary or other secondary renal diseases before mercury exposure were excluded, including systemic lupus erythematosus (SLE), infectious disease (especially hepatitis B and C virus) and other toxicant exposure. Clinical and laboratory data were recorded on admission as well as during follow-up. The determination of mercury intoxication was defined according to the diagnostic criteria for occupational mercury poisoning (GBZ89–2002) issued by the government of China, in which, the cut-off value of urinary mercury concentration was at least four times higher than that of the upper limit of 95th percentile of the U.S. population from the National Health and Nutrition Examination Survey (NHANES) in 2019 [7].

This research was in compliance with the Declaration of Helsinki and approved by the ethics committee of our hospital. Informed consent was obtained from the participants for sampling tissue and blood.

### Data collection

#### Quantification of mercury

The quantification of mercury in blood and urine was detected in the Poison Control Center, Affiliated Hospital of Military Medical Science Academy of the People’s Liberation Army by using the inductively coupled plasma mass spectrometry (ICP-MS) [8]. The reference value was < 2.5 μg/L.

#### Anti-PLA2R antibody in plasma detection

Plasma anti-PLA2R antibody level was detected by a commercial ELISA assay (EA 1254; EUROIMMUN AG, Lübeck, Germany), as reported previously [9]. The level > 20RU/ml was defined as positive.

### Renal histopathology

Percutaneous renal biopsy was performed using a semi-automatic biopsy gun under the guidance of ultrasound. Renal specimens were evaluated by direct immunofluorescence (IF), light microscopy and electron microscopy, according to the standard procedure in our hospital reported previously [10, 11].

### Glomerular-IgG subclasses distribution

Renal biopsy sections were formalin-fixed, paraffin-embedded, and cut into 4 μm for immunohistochemical staining. The sections were utilized with mouse monoclonal antibodies to human IgG1, IgG2, IgG3 and IgG4 (clone no. 4E3, HP6014, HP6050, HP6025; Southern Biotech, Birmingham, AL), the detection was performed with the method described previously [10]. Phosphate buffer saline (PBS) replacement of primary antibodies was used as the negative control. Normal renal tissues far from renal carcinoma were used as the healthy control. The intensity of glomerular staining was semiquantitatively graded as negative (score 0), weak positive or partial positive along the glomerular basement membrane (GBM) (score 1), moderate positive or continuous positive along the GBM (score 2), strong positive (score 3). The scoring was performed by two independent researchers who were blinded to clinical data. The results were expressed as the frequency of positive biopsies (percentage) and the score (mean rank) of deposition of each IgG subclass.

### Detection of glomerular PLA2R expression

Paraffin-embedded sections of formalin-fixed renal tissue were utilized for immunohistochemistry to detect the expression of glomerular PLA2R, as reported previously [12]. PBS replaced the primary antibodies as negative controls and normal kidney tissues far from the renal carcinoma were used as healthy controls. Positivity of glomerular PLA2R expression was defined as linear or granular diffuse staining on glomeruli.

### Treatment

The main treatment included removal of exposure and chelation for mercury detoxification. Corticosteroids and immunosuppressive agents, including cyclophosphamide and cyclosporine A, were prescribed in those unresponsive to chelation therapy.

The specific regimen of mercury detoxification was an intramuscular injection (0.125~0.250 g/d) of sodium dimercaptosulfonate (DMPS) for 3 consecutive days followed by a 4-day interval [13, 14]. The regimen continued until the urine mercury level dropped to the normal range or severe side-effects occurred.

Thirty-four patients received mercury detoxification, which was recorded in detail, including the drug, rounds, the blood and urinary concentration during treatment, urinary excretion of protein, etc. The criteria of renal outcome were defined as follows [15]: *(1)* complete remission: the symptom improved remarkably, and reduction of proteinuria to < 0.3 g/d; *(2)* partial remission: the symptoms improved, and reduction of proteinuria to 0.3–3.5 g/d with ≥50% reduction compared with baseline; *(3)* treatment failure: no improvement in symptoms, or the reduction of proteinuria did not reach the abovementioned criteria.

### Statistical analysis

Statistical software SPSS 20.0 (IBM SPSS Statistics) was employed for statistical analysis. Chi-square analysis or Fisher’s exact test was used for comparison of categorical variables as appropriate. Continuous variables with a normal distribution were expressed as the mean ± SD and were compared using an independent-samples t test. Continuous variables with non-normal distributions were presented as the median with range (minimum, maximum) and were analyzed using the Mann-Whitney U test. Statistical significance was considered as *P* < 0.05.

## Results

### Demographic data of the patients and clinical presentation

There were 35 patients diagnosed as mercury-associated glomerulonephritis during the 6 years period in our hospital, accounting for 0.25% among the corresponding period of patients received native renal biopsy. The demographic data and clinical parameters of the 35 patients are shown in Table 1. Six patients were male, 29 were female, with an age of 36.1 ± 8.6 years at diagnosis. The duration of mercury exposure ranged from 1 to 120 months. The urinary mercury concentrations ranged from 10.0 to 138.2 μg/L. Twenty-two (62.9%) patients had nephrotic syndrome. All patients presented with proteinuria and normal renal function. The urinary protein ranged from 1.6 to 19.7 g/24 h.

**Table 1:** Demographic and clinical parameters of the 35 patients

	*N* = 35
Gender(male/female)	6/29
Age(years)	36.1 ± 8.6 (20~52)
Duration of Mercury Exposure, mo	5.0 (1.0~120.0)
Urinary Mercury Concentration, μg/L	29.6 (10.0~138.2)
Blood Mercury Concentration, μg/L	23.8 (8.2~180.0)
Edema, n/N (%)	28/35 (80.0%)
Nephrotic Syndrome, n/N (%)	22/35 (62.9%)
Hypertension, n/N (%)	6/36 (16.7%)
Urinary Protein (g/24 h)	4.6 (1.6~19.7)
Albumin (g/L)	20.2 (12.5~41.4)
Serum Creatinine (μmol/L)	62.9 ± 14.3 (32.3~90.0)
Microscopic Hematuria, n/N (%)	11/35 (31.4%)

Mercury exposure was caused by skin lighting cream in 20 patients (57.2%), mercury-containing pills in 9 patients (25.7%), hair-dyeing agents in 4 patients (11.4%), and unidentified reasons in 2 patients (5.7%). The mercury concentration of cream of one patient was detected, and it was up to 19,601 mg/kg, while the national quality standard was 1 mg/kg.

### Renal histopathology

Renal biopsies were performed in all patients. The most common renal histopathological pattern was MCD (21 patients, 60.0%), followed by MN (13 patients, 37.1%) and FSGS (1 patient, 2.9%).

**Fig. 1:**
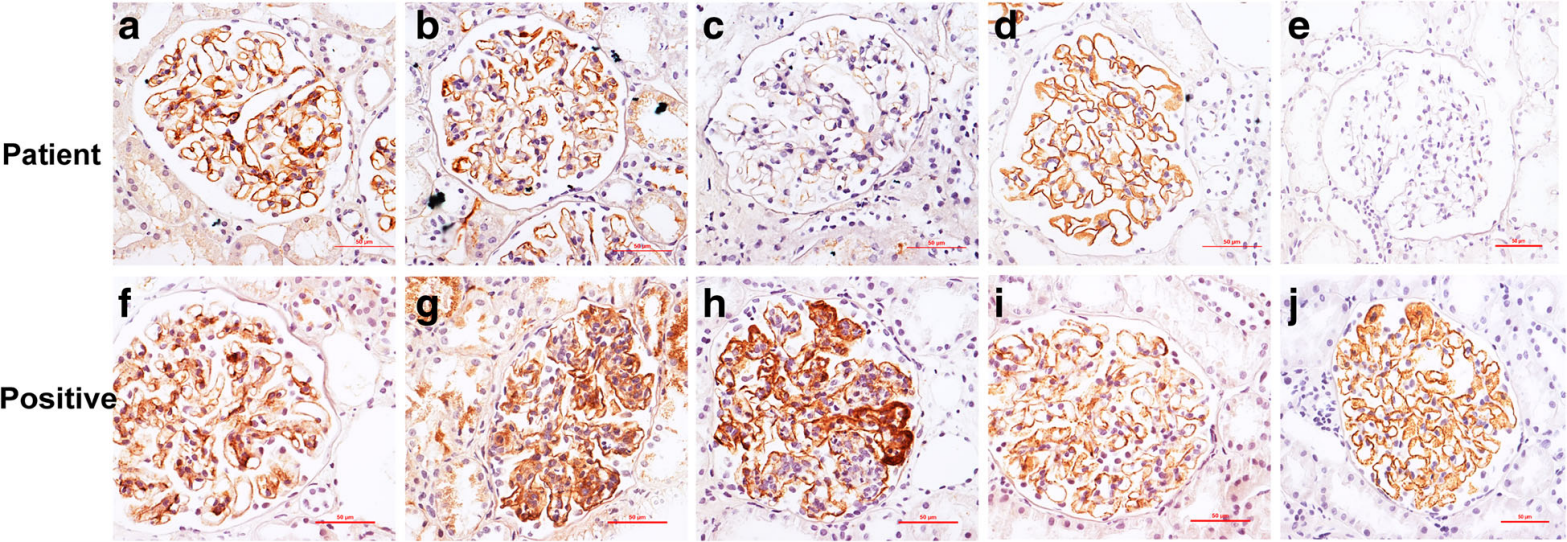
Immunohistochemical staining of glomerular IgG subclasses and PLA2R distribution (× 400). **a–e** are IgG1, IgG2, IgG3, IgG4 and PLA2R staining on the renal sections of a patient with mercury-associated MN, and the corresponding semi-quantitative scores are 2, 1, 0, 2 and 0, respectively. **f–j** are positive controls. **f**, **i** and **j** are IgG1, IgG4 and PLA2R staining on the renal sections of a patient with I-MN, and the corresponding semi-quantitative scores are 3, 2 and 3. **g** and **h** are IgG2 and IgG3 staining on the renal sections of a patient with systemic lupus erythematosus, and the corresponding semi-quantitative scores are 2 and 2

Glomerular IgG subclasses distribution and PLA2R along GBM in the 13 cases with MN were shown in Fig. 1 and Table 2. The anti-PLA2R antibody levels in plasma were listed in Table 2. There was a significant difference between the deposition frequency of IgG1 (61.5%), IgG2 (38.4%), IgG3 (0) and IgG4 (46.2%) deposition (*P* = 0.004), with predominant IgG1 deposition. Regarding the score of each IgG subclass deposition, the distribution of each IgG subclass was significantly different (H = 10.993, *P* = 0.012). The mean rank of IgG1 to IgG4 was 32.08, 26.04, 17.00 and 30.88, respectively, with the highest score of IgG1. All patients presented negative for plasma anti-PLA2R antibody and glomerular PLA2R antigen. According to the Ehrenreich and Churg’s classification criteria [16], 11 patients (84.6%) were classified as stage I MN.

**Table 2:** Distribution and intensity of IgG subclasses and PLA2R along GBM and anti-PLA2R in plasma of mercury-associated MN

Case	Gender	Age,yr	Histology	Glomeruli					Plasma
				IgG1	IgG2	IgG3	IgG4	PLA2R	Anti-PLA2R (RU/ml)
1	1	40~50	I-MN	0	0	0	0	0	2.46
2	1	30~40	I-MN	1	0	0	3	0	0
3	1	50~60	I-MN	1	1	0	1	0	0
4	1	30~40	I-MN	0	0	0	0	0	0
5	1	15~25	I-MN	0	0	0	1	0	1.06
6	1	35~45	I-MN	2	1	0	2	0	0
7	1	35~45	I-MN	1	1	0	0	0	1.06
8	1	30~40	I-MN	1	1	0	3	0	0.68
9	1	20~30	II-MN	1	0	0	3	0	2.10
10	2	40~50	I-MN	0	1	0	0	0	0
11	2	40~50	I-MN	0	0	0	0	0	0
12	1	35~45	I-MN	1	0	0	0	0	0
13	2	40~50	II-MN	1	0	0	0	0	2.33
Total				8	5	0	6		

*Abbreviations: PLA2R* M-type phospholipase A2 receptor, *GBM* glomerular basement membrane, *MN* membranous nephropathy

### Comparison between patients with MCD and MN

As shown in Table 3, there was no significant difference in age between patients with MCD and MN (35.1 ± 8.2 versus 38.7 ± 8.5 years, *P* = 0.223). Patients with MCD had significantly lower levels of serum albumin [18.5 (range 12.5~30.1) versus 23.9 (range 15.0~41.4) g/L, *P* = 0.011]. However, there was no significant difference in urinary protein between the two groups [6.0 (range 2.1~19.7) versus 3.7 (range 1.6~13.4) g/24 h, *P* = 0.130]. Furthermore, patients with MCD had significant shorter duration of mercury exposure than patients with MN [4.0 (range 1.0~120.0) versus 9.5 (range 2.0~120.0) months, *P* = 0.042]. However, the urinary mercury concentration of the former was significantly higher than that of the latter [36.2 (range 13.6~138.2) versus 22.2 (range 10.0~125.0) μg/L, *P* = 0.037].

**Table 3:** Comparison between patients with MCD and MN

parameters	MCD (*n* = 21)	MN (*n* = 13)	*P*-value
Age (year)	35.1 ± 8.2 (21~50)	38.7 ± 8.5 (20~52)	0.223
Urinary Protein (g/24 h)	6.0 (2.1~19.7)	3.7 (1.6~13.4)	0.130
Albumin (g/L)	18.5 (12.5~30.1)	23.9 (15.0~41.4)	0.011*
Duration of Mercury Exposure (mo)	4.0 (1.0~120.0)	9.5 (2.0~120.0)	0.042*
Urinary Mercury Concentration (μg/L)	36.2 (13.6~138.2)	22.2 (10.0~125.0)	0.037*
Blood Mercury Concentration (μg/L)	24.3 (8.7~180.0)	18.7 (8.2~97.0)	0.161

*Abbreviation: MCD* minimal change disease, *MN* membranous nephropathy

To further investigate the relationship between renal pathology and urinary concentration of mercury, we divided the 34 patients (excepting 1 patient with FSGS) into three groups by urinary mercury concentration: 10~20 μg/L, 20~30 μg/L and > 30 μg/L, and it was found that the proportion of patients with MCD significantly increased along with urinary mercury concentration (*P* = 0.024).

### Treatment and outcomes

**Fig. 2:**
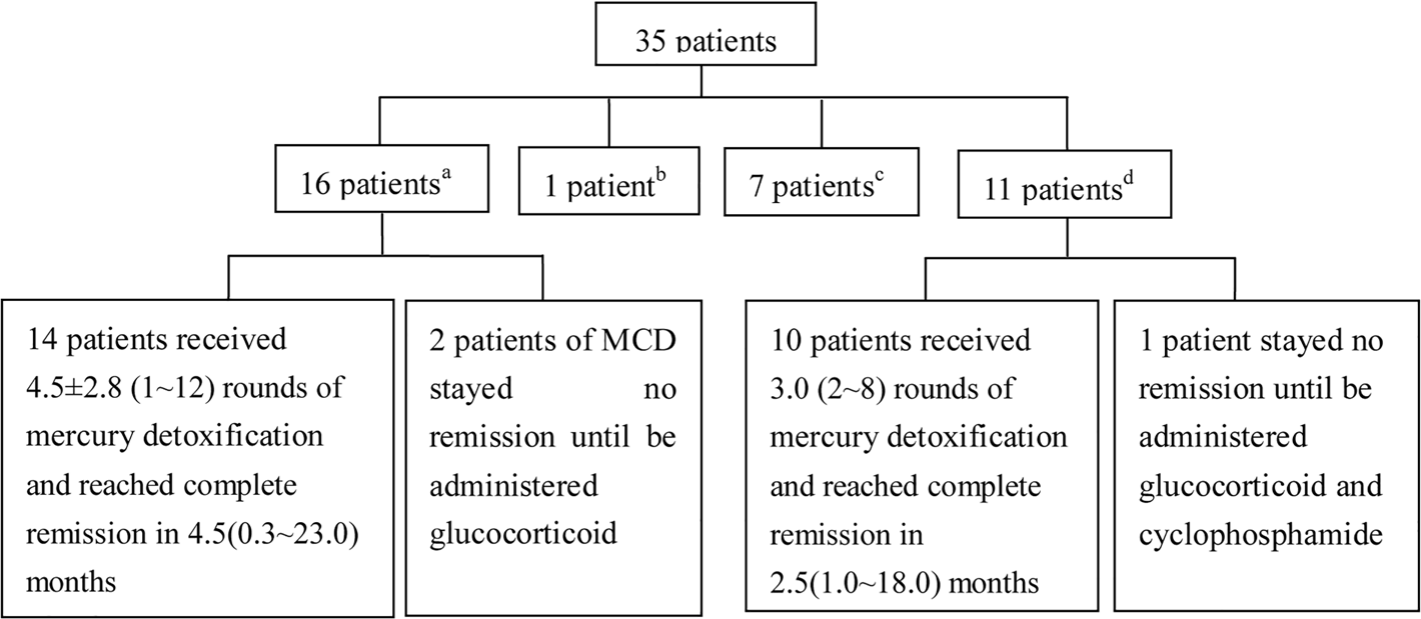
Specific therapeutic process of 35 patients of mercury-associated glomerulonephritis. ^a^no use of glucocorticoid or immunosuppressive agents before mercury detoxification. ^b^one patient of MN reached spontaneous complete remission without mercury detoxification. ^c^lost to follow-up (one of MN, five of MCD, and the other one of FSGS). ^d^stop use of glucocorticoid or immunosuppressive agents after definitive diagnosis and then begin mercury detoxification

All patients stopped contacting with mercury-containing preparations after establishment of the diagnosis. Treatment included mercury detoxification and a variety of other approach. Thirty-four patients received mercury detoxification of DMPS. The specific therapeutic process was listed in Fig. 2.

Sixteen patients (9 with MCD and 7 with MN) did not receive glucocorticoid or immunosuppressive agents before mercury detoxification. Among them, 14 patients (7 with MCD and 7 with MN) received 4.5 ± 2.8 (range 1~12) rounds of mercury detoxification and achieved complete remission in 4.5 (range 0.3~23.0) months, and 2 patients with MCD stayed no remission. The complete remission rate of mercury detoxification monotherapy was 87.5%. With the treatment of glucocorticoid, the other two patients achieved complete remission in 2 and 7 months, respectively.

Eleven patients (7 with MCD and 4 with MN) received initially immunosuppression before the establishment of the diagnosis and then received detoxification therapy. Among these patients, 10 (7 with MCD and 3 with MN) received 3.0 (range 2~8) rounds of mercury detoxification and achieved complete remission in 2.5 (range 1.0~18.0) months, while one patient with MN was resistant to the treatment until glucocorticoid and cyclophosphamide were employed, and achieved complete remission in 19 months.

To investigate the effect of mercury detoxification in different pathological pattern, we compared the remission rate, rounds of mercury detoxification and remission duration of the patients with MCD and MN who received mercury detoxification monotherapy. The remission rate of patients with MCD and MN was 77.8 and 100%, respectively. No significant difference was observed regarding to rounds of mercury detoxification [4 (range 1~7) versus 5 (range 3~12), *P* = 0.475], or remission duration between patients with MCD and MN [2 (range 0.25~23) versus 5 (range 1~12) months, *P* = 0.275].

## Discussion

There were 35 patients diagnosed as mercury-associated glomerulonephritis from 2010 to 2015, accounting for 0.25% among the corresponding period of patients underwent native renal biopsy in our hospital. The present study, to the best of our knowledge, has the largest case series of mercury-associated glomerulonephritis in single center. Extensive epidemiological studies have confirmed the toxic effects of mercury-containing cosmetics [4, 17, 18]. In this study, the exposure of mercury mainly comes from mercury-containing skin lightening cream, followed by hairy dyes and mercury-containing Chinese traditional medicine. The urinary concentrations of mercury in our patients were 4 to 55 times higher than reference value for general populations.

All patients in our study had mercury exposure before the presence of proteinuria and none of them had any history of primary or other secondary renal disease, such as SLE, infectious disease or other toxicant exposure. Furthermore, the glomerular PLA2R antigen, almost as a biomarker of idiopathic MN [19], was negative in all patients with mercury-associated MN. Therefore, glomerulonephritis probably resulted from mercury poisoning.

With regard to renal pathological pattern of mercury-associated glomerulonephritis, reports (including Chinese literature) were searched using PubMed (between 1962 and 2017) with mercury, mercury compounds, mercury poisoning, cosmetics and skin lightening cream as the subject headings, limited to human studies. Forty relevant reports consisting of 97 patients underwent renal biopsy were collected, and some major reports were listed in Table 4. The most common renal pathological pattern reported in the literature was MN, accounting for 71.1%, followed by MCD, accounting for 26.8%. However, in our study, MCD was the most common pattern, accounting for 60.0%. Mercury-associated MCD was first reported by Tang et al. in 2006 of a patient using mercury-containing skin lightening cream [20], and then be reported increasingly. Furthermore, we found the proportion of patients with MCD increased along with the increase of urinary mercury concentration. The different severity of mercury intoxication may be the reason for the different spectrum of mercury-associated glomerulonephritis among different regions, which needs to be investigated in multicenter studies.

**Table 4:** Summary of renal histopathology in systematic review of mercury-induced glomerulonephritis

Histology	Authors	No.
Total patients with biopsies		97
MN		69 (71.1%)
Membranous nephropathy after use of UK-manufactured skin creams containing mercury	Chakera et al. (2011) [18]	2
Mercury-induced membranous nephropathy: clinical and pathological features	Li et al. (2010) [6]	11
Membranous nephropathy from exposure to mercury in the fluorescent-tube recycling industry	Aymaz et al. (2001) [21]	2
Nephrotic syndrome after contact with mercury. A report of five cases, three after the use of ammoniated mercury ointment	Becker et al. (1962) [22]	5
MCD		26 (26.8%)
Nephrotic syndrome of minimal change diseasefollowing exposure to mercury-containingskin-lightening cream	Zhang et al. (2014) [14]	1
Mercury-induced nephrotic syndrome: a case report and review of the literature	Wagrowska-Danilewicz et al. (2014) [23]	1
Minimal change disease caused by exposure to mercury-containing skin lightening cream: a report of 4 cases	Tang et al. (2013) [17]	4
Minimal-change nephrotic syndrome due to occupational mercury vapor inhalation	Campbell et al. (2009) [24]	1
FSGS		2 (2.1%)
Mercury-Associated Nephrotic Syndrome: A Case Report and Systematic Review of the Literature	Miller et al. (2013) [25]	1

*Abbreviations: MN m*embranous nephropathy, *MCD* minimal change disease, *FSGS* focal segmental glomerular sclerosis

The mechanism of mercury-associated glomerulonephritis has not been fully elucidated. Immune mechanism plays an important role. It was suggested that mercury components have immunomodulating activity [26] and the nephrotic syndrome developed after mercury exposure results from idiosyncratic reactions [27] or an abnormal immune response to the heavy metal [2]. Studies demonstrated that low doses of mercury in susceptible rat strains induced an autoimmune syndrome with polyclonal B and T cell activation, increased serum IgG1 and IgE, anti-DNA, antiphospholipid, anti-glomerular basement membrane, antilaminin 1 and antithyroglobulin [28–30]. We speculated that high concentration of mercury directly damage the podocytes and cause MCD while long exposure to low concentration of mercury may cause MN through immune mechanism. The pathogenesis of MCD in mercury-associated glomerulonephritis required further investigation.

In patients with mercury-associated MN, IgG1 and IgG4 (predominantly IgG1) deposits were noted along the glomerular capillary loops, which was consistent with the study by Li et al. [6]. This distribution of IgG subclasses differed from idiopathic MN with predominant deposits of IgG4 [31], which was related to the immune response of Th2 response [32]. The both deposits of IgG1 and IgG4 in mercury-associated MN indicated that both Th1 and Th2 response were involved in the immune response [6].

Detoxification monotherapy is an effective therapy in mercury-associated glomerulonephritis. Among the 16 patients received mercury detoxification monotherapy, 87.5% of whom reached complete remission. The reason for the unresponsiveness to chelation therapy might be the inadequate rounds due to acute kidney injury after one round of therapy in one patient and discontinuation of mercury detoxification after two rounds without improvement in clinical conditions in another patient.

It should be noted that one patient of mercury-associated MN achieved spontaneous complete remission without mercury detoxification. Mercury can be eliminated by the body mainly through urine and feces, with the half-life about 2 months [1]. We speculated that after stopping mercury exposure, the immune response activated by mercury will be weakened along with the excretion of mercury, and spontaneous remission can be achieved. However, the remission time of this patient was 21 months, which was much longer than that of those who received mercury detoxification treatment (4.5 months). So mercury detoxification treatment is crucial. Additionally, there was a patient did not achieve remission until receiving steroids and cyclophosphamide (Fig. 2). This patient received a variety of drugs for psoriasis for 36 months before the onset of mercury-associated MN. We speculated that he had been exposed to mercury for a long time, and repeated antigen stimulation may cause sustained immune response, so mercury detoxification monotherapy was not enough for him, and steroids and immunosuppressive therapy were needed.

There are some limitations of the present study. First, it was a retrospective study, we did not routinely screen urinary mercury concentration of the patients, so the prevalence of mercury-associated glomerulonephritis may be underestimated. Second, due to the lost follow-up of seven patients, there might be some unresponsive bias in the analysis of prognosis.

## Conclusion

MCD was the most common renal histopathological pattern of mercury-associated glomerulonephritis, followed by MN. The proportion of MCD increased along with the increase of urinary mercury concentration. Because most patients can achieve remission by mercury detoxification therapy, it is important for clinicians to be aware of the possibility of mercury-associated glomerulonephritis, especially in patients with MCD and MN.

## Data Availability

The datasets used and/or analyzed during the current study are available from the corresponding author on reasonable request.

## Abbreviations

DMPS: Sodium dimercaptosulfonate
FSGS: Focal segmental glomerular sclerosis
GBM: Glomerular basement membrane
ICP-MS: Inductively coupled plasma mass spectrometry
IF: Immunofluorescence
MCD: Minimal change disease
Meth: Methenamine
MN: Membranous nephropathy
NHANES: the National Health and Nutrition Examination Survey
PBS: Phosphate buffer saline
PLA2R: M-type phospholipase A2 receptor
SLE: Systemic lupus erythematosus
